# Supplementary material for: A toolbox of IgG subclass-switched recombinant monoclonal antibodies for enhanced multiplex immunolabeling of brain
Source: eLife. 2019 Jan 22;8:e43322. doi: 10.7554/eLife.43322 (PMC6377228; doi:10.7554/eLife.43322)
Supplement: Supplementary file 1. — Table details for a set of 181 projects (130 successful, 51 unsuccesful) the number of colony PCR or restriction digest positive clones that were input into the COS-ICC validation assay, and the number and percentage of these that were positive in that assay. [file elife-43322-supp1.docx]

**Supplementary Table 1. Conversion efficiency of a selected subset of R-mAbs**

| **Successful Projects**  **(130 total)** | | | |  | **Unsuccessful Projects (51 total)** | |
| --- | --- | --- | --- | --- | --- | --- |
| **BciVI-resistant (129 total)** | | | |  | **BciVI-resistant** | |
| **mAb** | **Colony PCR/Restriction Digest Positives** | **COS-ICC Positives** | **% Positive** |  | **mAb** | **Colony PCR/Restriction Digest Positives** |
| D3/71 | 12 | 3 | 25.0 |  | K67/25 | 2 |
| K7/45 | 3 | 1 | 33.3 |  | K73/20 | 1 |
| K13/31 | 3 | 1 | 33.3 |  | K78/29 | 3 |
| K14/16 | 1 | 1 | 100.0 |  | L23/27 | 3 |
| K19/36 | 4 | 3 | 75.0 |  | L24/18 | 8 |
| K20/78 | 6 | 2 | 33.3 |  | L48A/9 | 1 |
| K25/73 | 8 | 5 | 62.5 |  | L57/46 | 3 |
| K28/86 | 1 | 1 | 100.0 |  | L58A/6 | 3 |
| K36/15 | 4 | 2 | 25.0 |  | L60/6 | 2 |
| K37/89 | 2 | 1 | 50.0 |  | L71/5 | 5 |
| K39/25 | 5 | 2 | 40.0 |  | L76/36 | 5 |
| K47/42 | 1 | 1 | 100.0 |  | L92/69 | 6 |
| K51/1 | 2 | 1 | 50.0 |  | L92/70 | 10 |
| K55/7 | 5 | 3 | 60.0 |  | L92/89 | 9 |
| K56A/50 | 4 | 3 | 75.0 |  | N18/28 | 5 |
| K57/1 | 4 | 2 | 50.0 |  | N38/8 | 6 |
| K64/15 | 4 | 3 | 75.0 |  | N64A/36 | 1 |
| K66/38 | 4 | 3 | 75.0 |  | N68/6 | 15 |
| K72/8 | 3 | 1 | 33.3 |  | N71/37 | 4 |
| K75/41 | 5 | 1 | 20.0 |  | N81/2 | 5 |
| K91/36 | 7 | 3 | 42.9 |  | N86/8 | 2 |
| K96/7 | 2 | 2 | 100.0 |  | N86/44 | 4 |
| L5/1 | 1 | 1 | 100.0 |  | N87/25 | 3 |
| L8/15 | 1 | 1 | 100.0 |  | N92/14 | 4 |
| L18A/3 | 7 | 2 | 28.6 |  | N95/35 | 2 |
| L20/8 | 10 | 1 | 10.0 |  | N97A/31 | 2 |
| L21/32 | 11 | 7 | 63.6 |  | N105/17 | 5 |
| L42/17 | 11 | 4 | 36.4 |  | N119/44 | 1 |
| L43/40 | 6 | 2 | 33.3 |  | N121A/1 | 2 |
| L45/30 | 9 | 1 | 11.1 |  | N125/10 | 2 |
| L51/82 | 2 | 1 | 50.0 |  | N129/15 | 3 |
| L60/4 | 8 | 3 | 37.5 |  | N134/12 | 3 |
| L61/14 | 4 | 2 | 50 |  | N141/28 | 1 |
| N2/16 | 4 | 3 | 75.0 |  | N142/28 | 5 |
| N3/26 | 8 | 1 | 12.5 |  | N144/17 | 2 |
| N6/38 | 4 | 1 | 25.0 |  | N144/32 | 3 |
| N7/18 | 5 | 3 | 60.0 |  | N170A/26 | 3 |
| N11/33 | 13 | 2 | 15.4 |  | N173B/13 | 4 |
| N15/4 | 2 | 1 | 50.0 |  | N183/15 | 1 |
| N16B/8 | 3 | 3 | 100.0 |  | N209C/35 | 3 |
| N18/30 | 11 | 3 | 27.3 |  | N212A/34 | 1 |
| N23B/49 | 2 | 2 | 100.0 |  | N308/48 | 6 |
| N28/9 | 7 | 4 | 57.1 |  | N319/14 | 1 |
| N29/29 | 8 | 4 | 50.0 |  | N326D/13 | 2 |
| N34/34 | 3 | 1 | 33.3 |  | N330A/80 | 2 |
| N50/36 | 5 | 1 | 20.0 |  | N356/9 | 2 |
| N51/6 | 4 | 2 | 50.0 |  | N363/71 | 2 |
| N52A/42 | 9 | 4 | 44.4 |  | N388A/27 | 3 |
| N59/36 | 5 | 1 | 20.0 |  | N399/19 | 2 |
| N70/28 | 3 | 1 | 33.3 |  | N408/79 | 1 |
| N77/15 | 5 | 1 | 20.0 |  | N422/18 | 4 |
| N81/37 | 6 | 4 | 66.7 |  | **Total** | **178** |
| N83/48 | 4 | 1 | 25.0 |  |  |  |
| N84/37 | 6 | 4 | 66.7 |  |  |  |
| N85/18 | 5 | 2 | 40.0 |  |  |  |
| N85/37 | 6 | 4 | 66.7 |  |  |  |
| N86/38 | 4 | 2 | 50.0 |  |  |  |
| N87/25 | 4 | 2 | 50.0 |  |  |  |
| N88/12 | 6 | 5 | 83.3 |  |  |  |
| N93A/49 | 6 | 1 | 16.7 |  |  |  |
| N94/17 | 6 | 2 | 33.3 |  |  |  |
| N96/55 | 6 | 5 | 83.3 |  |  |  |
| N104/32 | 5 | 2 | 40.0 |  |  |  |
| N110/29 | 5 | 2 | 40.0 |  |  |  |
| N111/24 | 3 | 1 | 33.3 |  |  |  |
| N112B/14 | 4 | 1 | 25.0 |  |  |  |
| N116/14 | 7 | 3 | 42.9 |  |  |  |
| N117/9 | 8 | 5 | 62.5 |  |  |  |
| N121A/31 | 6 | 4 | 66.7 |  |  |  |
| N124B/38 | 3 | 2 | 66.7 |  |  |  |
| N127/31 | 9 | 8 | 88.9 |  |  |  |
| N129A/6 | 6 | 2 | 33.3 |  |  |  |
| N133/21 | 4 | 2 | 50.0 |  |  |  |
| N135/37 | 7 | 1 | 14.3 |  |  |  |
| N147/6 | 3 | 2 | 66.7 |  |  |  |
| N164/6 | 2 | 1 | 50.0 |  |  |  |
| N168/6 | 4 | 1 | 25.0 |  |  |  |
| N170A/1 | 5 | 1 | 20.0 |  |  |  |
| N171/17 | 8 | 2 | 25.0 |  |  |  |
| N174B/27 | 3 | 3 | 100.0 |  |  |  |
| N176A/35 | 2 | 1 | 50.0 |  |  |  |
| N178A/9 | 4 | 2 | 50.0 |  |  |  |
| N180/41 | 3 | 2 | 66.7 |  |  |  |
| N195A/16 | 11 | 3 | 27.3 |  |  |  |
| N196/16 | 6 | 3 | 50.0 |  |  |  |
| N201/35 | 2 | 2 | 100.0 |  |  |  |
| N205B/22 | 2 | 1 | 50.0 |  |  |  |
| N206A/8 | 10 | 1 | 10.0 |  |  |  |
| N206B/9 | 7 | 5 | 71.4 |  |  |  |
| N212/3 | 7 | 4 | 57.1 |  |  |  |
| N212/7 | 4 | 1 | 25.0 |  |  |  |
| N212/17 | 8 | 4 | 50.0 |  |  |  |
| N222/6 | 11 | 4 | 36.4 |  |  |  |
| N229A/32 | 8 | 8 | 100.0 |  |  |  |
| N230/21 | 8 | 3 | 37.5 |  |  |  |
| N235/22 | 9 | 7 | 77.8 |  |  |  |
| N244/5 | 7 | 2 | 28.6 |  |  |  |
| N268/19 | 8 | 1 | 12.5 |  |  |  |
| N269/73 | 10 | 5 | 50.0 |  |  |  |
| N271/44 | 2 | 1 | 50.0 |  |  |  |
| N277/7 | 9 | 2 | 22.2 |  |  |  |
| N278/19 | 3 | 1 | 33.3 |  |  |  |
| N279B/27 | 10 | 10 | 100.0 |  |  |  |
| N289/16 | 3 | 3 | 100.0 |  |  |  |
| N291C/22 | 7 | 5 | 71.4 |  |  |  |
| N295B/54 | 5 | 2 | 40.0 |  |  |  |
| N295B/66 | 10 | 5 | 50.0 |  |  |  |
| N320/48 | 9 | 6 | 66.7 |  |  |  |
| N321C/49 | 2 | 1 | 50.0 |  |  |  |
| N323B/20 | 6 | 4 | 66.7 |  |  |  |
| N325B/65 | 4 | 1 | 25.0 |  |  |  |
| N326D/29 | 11 | 10 | 90.9 |  |  |  |
| N328B/37 | 10 | 2 | 20.0 |  |  |  |
| N331/19 | 10 | 3 | 30.0 |  |  |  |
| N341/37 | 6 | 2 | 33.3 |  |  |  |
| N346/9 | 2 | 2 | 100.0 |  |  |  |
| N348/82 | 8 | 4 | 50.0 |  |  |  |
| N353/13 | 6 | 2 | 33.3 |  |  |  |
| N355/1 | 7 | 4 | 57.1 |  |  |  |
| N356/23 | 10 | 7 | 70.0 |  |  |  |
| N359/28 | 7 | 6 | 85.7 |  |  |  |
| N388A/10 | 4 | 2 | 50.0 |  |  |  |
| N388A/60 | 8 | 1 | 12.5 |  |  |  |
| N398A/34 | 11 | 11 | 100.0 |  |  |  |
| N413/67 | 1 | 1 | 100.0 |  |  |  |
| N415/24 | 6 | 4 | 66.7 |  |  |  |
| N419/40 | 13 | 10 | 76.9 |  |  |  |
| N419/78 | 3 | 1 | 33.3 |  |  |  |
| 1F1 | 9 | 4 | 44.4 |  |  |  |
| **Total** | **741** | **359** | **48.4** |  |  |  |
|  |  |  |  |  |  |  |
| **BciVI-sensitive** | | | |  |  |  |
| K58/35 | 19 | 7 | 36.8 |  |  |  |
