# Supplementary material for: A toolbox of IgG subclass-switched recombinant monoclonal antibodies for enhanced multiplex immunolabeling of brain
Source: eLife. 2019 Jan 22;8:e43322. doi: 10.7554/eLife.43322 (PMC6377228; doi:10.7554/eLife.43322)
Supplement: Supplementary file 2. — Table lists the mAbs successfully converted to R-mAbs to date. For each mAb, the mAb clone number, the target protein, the mAb IgG subclass (in sentence case), the R-mAb IgG subclass (in upper case) and the original mAb and the cloned R-mAb RRID numbers in the Antibody Registry are detailed. [file elife-43322-supp2.docx]

**Supplementary Table 2. mAbs converted to validated R-mAbs**

| **Clone** | **Target** | **mAb IgG subclass** | **R-mAb IgG subclass** | **mAb RRID** | **R-mAb RRID** |
| --- | --- | --- | --- | --- | --- |
| 1F1 | TrpC1 | IgG1 | IgG2A | AB_10999751 | AB_2750650 |
| D3/71 | Kv2.1 K+ channel | IgG1 | IgG2A |  | AB_2750651 |
| K7/45 | Kv1.5 | IgG1 | IgG2A | AB_10675288 | AB_2750674 |
| K9/40 | Kvbeta1.1 K+ channel | IgG2b | IgG2A | AB_10672857 | AB_2750678 |
| K13/31 | Kv1.4 K+ channel | IgG1 | IgG2A | AB_10673576 | AB_2750652 |
| K14/16 | Kv1.2 K+ channel | IgG2b | IgG2A | AB_10674277 | AB_2750653 |
| K17/70 | Kvbeta2 K+ channel | IgG1 | IgG2A | AB_10673520 | AB_2750654 |
| K19/36 | Kv1.6 K+ channel | IgG3 | IgG2A | AB_10671825 | AB_2750655 |
| K20/78 | Kv1.1 K+ channel | IgG1 | IgG2A | AB_10672854 | AB_2750656 |
| K25/73 | Pan-Kvbeta K+ channel | IgG1 | IgG2A | AB_2336914 | AB_2750657 |
| K28/43 | PSD-95 | IgG2a | IgG1 | AB_10698024 | AB_2750658 |
| K28/43 | PSD-95 | IgG2a | IgG2A | AB_10698024 | AB_2750659 |
| K28/86 | Pan-MAGUK | IgG1 | IgG2A | AB_10698179 | AB_2750660 |
| K36/15 | Kv1.1 K+ channel (external) | IgG2b | IgG2A | AB_10673166 | AB_2750661 |
| K37/89 | Kv2.2 K+ channel | IgG2a | IgG2A | AB_10673393 | AB_2750662 |
| K39/25 | Kv2.1 K+ channel (external) | IgG2a | IgG2A | AB_10674578 | AB_2750663 |
| K40/17 | Pan-Kvbeta K+ channel | IgG2a | IgG2A | AB_10672417 | AB_2750664 |
| K47/42 | Kvbeta1.2 K+ channel | IgG1 | IgG2A | AB_10671827 | AB_2750665 |
| K51/1 | SH3GL1/Endophilin A2 | IgG1 | IgG2A | AB_10698030 | AB_2750666 |
| K55/7 | KChIP1 K+ channel | IgG1 | IgG2A | AB_10697876 | AB_2750667 |
| K55/82 | KChIP1 K+ channel | IgG2a | IgG2A | AB_2132595 | AB_2750668 |
| K56A/50 | CASK | IgG1 | IgG2A | AB_10671954 | AB_2750669 |
| K57/1 | Kv4.2 K+ channel (external) | IgG1 | IgG2A | AB_10672254 | AB_2750670 |
| K58/35 | pan-Nav | IgG1 | IgG2A | AB_477552 | AB_2750671 |
| K64/15 | SAP97 | IgG1 | IgG2a | AB_10672989 | AB_2750672 |
| K66/38 | KChIP3 K+ channel | IgG2a | IgG2a | AB_10697878 | AB_2750673 |
| K72/8 | Co-Rest/RCOR1 | IgG1 | IgG2A | AB_10673564 | AB_2750675 |
| K75/41 | Kv4.3 K+ channel | IgG1 | IgG2A | AB_10672856 | AB_2750676 |
| K89/34 | Kv2.1 K+ channel | IgG1 | IgG2A | AB_10672253 | AB_2750677 |
| K91/36 | WAVE1/SCAR | IgG1 | IgG2A | AB_10671317 | AB_2750679 |
| K96/7 | Pancortin | IgG1 | IgG2A | AB_10674112 | AB_2750680 |
| L5/1 | SCG10/Stathmin-2 | IgG2b | IgG2A | AB_10671663 | AB_2750688 |
| L6/23 | Slo1/BKAlpha maxi-K+ channel | IgG1 | IgG2A | AB_2493094 | AB_2750690 |
| L8/15 | Snapin/SNAPAP | IgG1 | IgG2A | AB_10675326 | AB_2750694 |
| L18A/3 | BKbeta4 K+ channel | IgG2a | IgG2A | AB_10675133 | AB_2750681 |
| L20/8 | PICK1 | IgG2a | IgG2A | AB_10672986 | AB_2750682 |
| L21/32 | GluA2/GluR2 glutamate receptor | IgG1 | IgG2A | AB_10674575 | AB_2750683 |
| L28/4 | Kv4.2 K+ channel | IgG1 | IgG2A | AB_2315873 | AB_2750684 |
| L42/17 | SynDIG1/Tmem90b | IgG2a | IgG2A | AB_11000166 | AB_2750685 |
| L43/40 | DLX1 | IgG2a | IgG2A | AB_10671701 | AB_2750686 |
| L45/30 | SynCAM1 | IgG1 | IgG2A | AB_10671665 | AB_2750687 |
| L51/82 | MMP9 | IgG2a | IgG2A | AB_10672974 | AB_2750689 |
| L60/4 | Copper ATPase 1 (Menke's disease protein) | IgG2b | IgG2A | AB_10672736 | AB_2750691 |
| L61/14 | Kv2.1 K+ channel pS603 | IgG1 | IgG2A | AB_2315769 | AB_2750692 |
| L62/29 | Copper ATPase 2 (Wilson's disease protein) | IgG1 | IgG2A | AB_10672737 | AB_2750693 |
| L80/21 | Kv2.1 K+ channel | IgG3 | IgG2A | AB_2315862 | AB_2750695 |
| N2/16 | KCNU1/Slo3 maxi-K+ channel | IgG1 | IgG2A | AB_10698181 | AB_2750743 |
| N3/26 | KCNT1/Slo2.2/Slack K+ channel | IgG1 | IgG2A | AB_10698032 | AB_2750772 |
| N6/38 | VAChT | IgG1 | IgG2A | AB_10674274 | AB_2750807 |
| N7/18 | Cavbeta1 Ca2+ channel | IgG2a | IgG2A | AB_10673098 | AB_2750809 |
| N8B/1 | Cavbeta2 Ca2+ channel | IgG1 | IgG2A | AB_10675135 | AB_2750822 |
| N10/7 | Cavbeta4 Ca2+ channel | IgG1 | IgG2A | AB_10671176 | AB_2750696 |
| N11/33 | KCNT2/Slo2.1/Slick K+ channel | IgG1 | IgG2A | AB_10675450 | AB_2750700 |
| N15/4 | TrpV3 | IgG2a | IgG2A | AB_10675455 | AB_2750722 |
| N15/39 | TrpV3 | IgG1 | IgG2A | AB_10675456 | AB_2750721 |
| N16B/8 | Kv3.1b K+ channel | IgG1 | IgG2A | AB_10672409 | AB_2750730 |
| N18/30 | PSD-93/Chapsyn-110 | IgG1 | IgG2A | AB_10673100 | AB_2750737 |
| N19/2 | SAP102 | IgG1 | IgG2A | AB_10671660 | AB_2750740 |
| N22/21 | Shank1 | IgG1 | IgG2A | AB_10673108 | AB_2750751 |
| N23B/6 | Shank2 | IgG1 | IgG2A | AB_10674119 | AB_2750757 |
| N23B/49 | Pan-Shank | IgG1 | IgG2A | AB_10674115 | AB_2750756 |
| N26A/23 | Kv7.2/KCNQ2 K+ channel | IgG1 | IgG2A | AB_10673164 | AB_2750761 |
| N28/9 | VGlut1 | IgG1 | IgG2A | AB_10673111 | AB_2750766 |
| N29/29 | VGlut2 | IgG1 | IgG2A | AB_10698039 | AB_2750768 |
| N34/34 | VGlut3 | IgG1 | IgG2A | AB_10698040 | AB_2750780 |
| N37A/10 | Kv7.1/KCNQ1 K+ channel | IgG1 | IgG2A | AB_10675286 | AB_2750788 |
| N40B/18 | BKbeta3a K+ channel | IgG1 | IgG2A | AB_10673961 | AB_2750792 |
| N49A/21 | NGL-1/LRRC4C | IgG1 | IgG2A | AB_10671309 | AB_2750797 |
| N50/36 | NGL-2/LRRC4 | IgG1 | IgG2A | AB_10697883 | AB_2750798 |
| N51/6 | NGL-3/LRRC4B | IgG1 | IgG2A | AB_10672415 | AB_2750799 |
| N52A/42 | Mortalin/GRP75 | IgG1 | IgG2A | AB_10674108 | AB_2750800 |
| N52B/27 | SALM2/LRFN1 | IgG1 | IgG2A | AB_10674118 | AB_2750801 |
| N53/32 | BKbeta2 K+ channel | IgG2a | IgG2A | AB_10674434 | AB_2750802 |
| N55/10 | Cav3.2 Ca2+ channel | IgG1 | IgG2A | AB_2315825 | AB_2750803 |
| N56/21 | FGF14/FHF4 | IgG1 | IgG2A | AB_10671703 | AB_2750804 |
| N57/2 | ADAM22 (extracellular) | IgG1 | IgG2A | AB_10671172 | AB_2750805 |
| N59/36 | GluN2B/NR2B glutamate receptor | IgG2b | IgG2a | AB_10672980 | AB_2750806 |
| N69/46 | Shank3 | IgG2b | IgG2a | AB_10698031 | AB_2750808 |
| N70/28 | HCN1 | IgG1 | IgG2A | AB_10672848 | AB_2750810 |
| N75/3 | mGluR1/5 (Group I) glutamate receptor | IgG2a | IgG2A | AB_10672304 | AB_2750811 |
| N76/3 | Ataxin-1, 11NQ | IgG1 | IgG2A | AB_10673969 | AB_2750812 |
| N76/8 | Ataxin-1, 11NQ | IgG2b | IgG2A | AB_10671173 | AB_2750813 |
| N77/15 | TrpC4 | IgG2b | IgG2A | AB_10698036 | AB_2750814 |
| N81/37 | GABA(B)R2 | IgG1 | IgG2A | AB_10672297 | AB_2750815 |
| N83/48 | GIT2 | IgG1 | IgG2A | AB_10672302 | AB_2750816 |
| N84/37 | Laforin | IgG1 | IgG2A | AB_10673395 | AB_2750817 |
| N85/18 | Malin | IgG1 | IgG2A | AB_10673396 | AB_2750818 |
| N86/38 | GFP | IgG2a | IgG2a | AB_10671955 | AB_2750819 |
| N87/25 | GABA(A)R, Beta3 | IgG1 | IgG2a | AB_10673389 | AB_2750820 |
| N88/12 | MESD | IgG2b | IgG2A | AB_10673280 | AB_2750821 |
| N91/27 | FGF13/FHF2 | IgG1 | IgG2A | AB_10675138 | AB_2750823 |
| N93A/49 | GABA(B)R1 | IgG1 | IgG2A | AB_10672843 | AB_2750824 |
| N94/17 | FGF12/FHF1 | IgG1 | IgG2A | AB_10675137 | AB_2750825 |
| N96/55 | GABA(A)R, Beta1 | IgG1 | IgG2A | AB_10673157 | AB_2750826 |
| N97A/31 | Neuroligin-1 | IgG1 | IgG2A | AB_10671307 | AB_2750827 |
| N98/7 | Neuroligin-4* | IgG1 | IgG2A | AB_2151948 | AB_2750828 |
| N103/39 | Aldh1L1 (blotting) | IgG1 | IgG2A | AB_10673447 | AB_2750697 |
| N104/32 | SNAT1 | IgG1 | IgG2A | AB_10674120 | AB_2750698 |
| N106/20 | Ankyrin-G (blotting) | IgG1 | IgG2A | AB_10674433 | AB_2750699 |
| N110/29 | Neuroligin-3 | IgG1 | IgG2A | AB_10673403 | AB_2750701 |
| N111/24 | Mitofusin-1 | IgG1 | IgG2A | AB_10675293 | AB_2750702 |
| N112B/14 | Kir2.1 K+ channel | IgG1 | IgG2A | AB_11001668 | AB_2750703 |
| N114/10 | HCN4 | IgG1 | IgG2A | AB_10673158 | AB_2750704 |
| N116/14 | Botch | IgG1 | IgG2A | AB_10671697 | AB_2750705 |
| N117/9 | DNMT3L | IgG1 | IgG2A | AB_10673155 | AB_2750706 |
| N120A/9 | Neuregulin-HBD (Heparin binding domain, Type I/II) | IgG2a | IgG2A | AB_2154679 | AB_2750707 |
| N121A/31 | Pannexin-2 | IgG1 | IgG2A | AB_10673509 | AB_2750708 |
| N123/19 | Histone H3-pThr11 | IgG1 | IgG2A | AB_10673569 | AB_2750709 |
| N124B/38 | Kir2.2 K+ channel | IgG1 | IgG2A | AB_10697879 | AB_2750710 |
| N127/31 | Pan-SAPAP | IgG2b | IgG2A | AB_10697886 | AB_2750711 |
| N129A/6 | Mad3 | IgG2b | IgG2A | AB_10674581 | AB_2750712 |
| N133/21 | RGS14 | IgG2a | IgG2A | AB_10698026 | AB_2750713 |
| N135/37 | JIP-2/IB-2 | IgG1 | IgG2A | AB_10672307 | AB_2750714 |
| N138/6 | LRRK2/Dardarin | IgG1 | IgG2A | AB_10697869 | AB_2750715 |
| N144/14 | 6xHis | IgG1 | IgG2A | AB_10671171 | AB_2750716 |
| N144/17 | Gamma-protocadherin-A3 | IgG1 | IgG2A | AB_10697874 | AB_2750717 |
| N145/20 | Pan-GRK | IgG1 | IgG2A | AB_10671656 | AB_2750718 |
| N147/6 | Pan-QKI | IgG2b | IgG2A | AB_10671658 | AB_2750719 |
| N149/25 | Olig1 | IgG1 | IgG2A | AB_10674111 | AB_2750720 |
| N150/21 | Doc2b | IgG1 | IgG2A | AB_2277497 | AB_2750723 |
| N151/3 | GABA(A)R, Delta | IgG2a | IgG2A | AB_10672295 | AB_2750724 |
| N153/5 | Mitofusin-2 | IgG2a | IgG2A | AB_10672973 | AB_2750725 |
| N155/9 | Uncx | IgG1 | IgG2A | AB_10672528 | AB_2750726 |
| N160/21 | SynDIG3/Tmem91 | IgG2b | IgG2A | AB_10676102 | AB_2750727 |
| N164/6 | LRP4 (cytoplasmic) | IgG1 | IgG2A | AB_10672256 | AB_2750728 |
| N168/6 | Navbeta4 Na+ channel | IgG1 | IgG2A | AB_10673578 | AB_2750729 |
| N170A/1 | Neurexin-1-Beta | IgG1 | IgG2A | AB_10672978 | AB_2750731 |
| N170A/26 | Neurexin-1-Beta | IgG1 | IgG2A | AB_10674585 | AB_2750732 |
| N171/17 | INPP4b | IgG1 | IgG2A | AB_2296057 | AB_2750733 |
| N174B/27 | Gamma-protocadherin-C3 | IgG1 | IgG2A | AB_10675141 | AB_2750734 |
| N176A/35 | S100A5 | IgG1 | IgG2A | AB_2183799 | AB_2750735 |
| N178A/9 | Cav3.1 Ca2+ channel | IgG1 | IgG2A | AB_10673097 | AB_2750736 |
| N180/41 | EAAC1 | IgG1 | IgG2A | AB_10697871 | AB_2750738 |
| N182/17 | QKI-6 | IgG1 | IgG2A | AB_10673511 | AB_2750739 |
| N195A/16 | QKI-5 | IgG1 | IgG2A | AB_10676099 | AB_2750741 |
| N196/16 | PARIS/ZNF746 | IgG1 | IgG2A | AB_10675299 | AB_2750742 |
| N201/35 | Iduna/RNF146 | IgG1 | IgG2A | AB_10675284 | AB_2750744 |
| N205B/22 | LRRTM4 | IgG1 | IgG2A | AB_10674105 | AB_2750745 |
| N206A/8 | GFAP | IgG1 | IgG2A | AB_10672298 | AB_2750746 |
| N206B/9 | GFAP R416WT | IgG1 | IgG2A | AB_11000181 | AB_2750747 |
| N212/17 | TRIP8b (constant) | IgG2a | IgG2A | AB_10675453 | AB_2750748 |
| N212/3 | TRIP8b (exon 4) | IgG1 | IgG2A | AB_2162409 | AB_2750749 |
| N212/7 | TRIP8b (constant) | IgG2b | IgG2A | AB_10698034 | AB_2750750 |
| N222/6 | Clcn4 | IgG1 | IgG2A | AB_11001829 | AB_2750752 |
| N229A/32 | GABA(A)R, Alpha6 | IgG1 | IgG2A | AB_2336906 | AB_2750753 |
| N230/21 | MMACHC | IgG1 | IgG2A | AB_10673282 | AB_2750754 |
| N235/22 | Pan-FHF-A | IgG2b | IgG2A | AB_10672416 | AB_2750755 |
| N244/5 | SynCAM4 | IgG1 | IgG2A | AB_10673109 | AB_2750758 |
| N268/19 | TRPML3/Mucolipin-3 | IgG2a | IgG2A | AB_11000172 | AB_2750759 |
| N269/73 | Synaptotagmin-10 | IgG1 | IgG2A | AB_10672424 | AB_2750760 |
| N271/44 | ASIC1 | IgG1 | IgG2A | AB_11000719 | AB_2750762 |
| N277/7 | Synaptotagmin-12 | IgG1 | IgG2A | AB_2315939 | AB_2750763 |
| N278/19 | Synaptotagmin-3 | IgG1 | IgG2A | AB_11001828 | AB_2750764 |
| N279B/27 | GluK5/Grik5/KA2 kainate receptor | IgG2a | IgG2A | AB_2315855 | AB_2750765 |
| N289/16 | SUR1 | IgG1 | IgG2A | AB_11001671 | AB_2750767 |
| N291C/22 | TRIP8b (exon 1a/5) | IgG1 | IgG2A | AB_2315949 | AB_2750769 |
| N295B/54 | Arl13b | IgG2b | IgG2A |  | AB_2750770 |
| N295B/66 | Arl13b | IgG2a | IgG2A | AB_11000053 | AB_2750771 |
| N320/48 | Kirrel3, short isoform 3 | IgG2a | IgG2A | AB_11030249 | AB_2750773 |
| N321C/49 | Kirrel3, short and long | IgG2b | IgG2A | AB_2315856 | AB_2750774 |
| N323B/20 | SUR2B | IgG2b | IgG2A | AB_2341102 | AB_2750775 |
| N325B/65 | THAP1 | IgG3 | IgG2A | AB_2315943 | AB_2750776 |
| N326D/29 | REEP1/2 | IgG1 | IgG2A | AB_2315914 | AB_2750777 |
| N328B/37 | ZNF423 | IgG1 | IgG2A | AB_2315960 | AB_2750778 |
| N331/19 | Dopamine D3 receptor | IgG2a | IgG2A | AB_2315831 | AB_2750779 |
| N341/37 | LRRK1 | IgG2a | IgG2A | AB_2315877 | AB_2750781 |
| N346/9 | Stonin-2 | IgG2b | IgG2A | AB_2315923 | AB_2750782 |
| N348/82 | NSD3 | IgG1 | IgG2A | AB_2315901 | AB_2750783 |
| N353/13 | Flrt3 | IgG1 | IgG2A | AB_2315832 | AB_2750784 |
| N355/1 | GluA1/GluR1 glutamate receptor | IgG1 | IgG2A | AB_2315839 | AB_2750785 |
| N356/23 | SVOP | IgG1 | IgG2A | AB_2315933 | AB_2750786 |
| N359/28 | Foxi3 | IgG1 | IgG2A | AB_2315836 | AB_2750787 |
| N388A/10 | Ankyrin-R | IgG2b | IgG2A | AB_2336901 | AB_2750789 |
| N388A/60 | Pan-Ankyrin | IgG1 | IgG2A | AB_2336913 | AB_2750790 |
| N398A/34 | GABA(A)R, Alpha4 | IgG1 | IgG2A | AB_2336905 | AB_2750791 |
| N413/67 | GABA(A)R, Epsilon | IgG2a | IgG2A | AB_2341105 | AB_2750793 |
| N415/24 | GABA(A)R, Alpha5 | IgG1 | IgG2A | AB_2491075 | AB_2750794 |
| N419/40 | Pan-Nav1 Na+ channel | IgG2a | IgG2A | AB_2491079 | AB_2750795 |
| N419/78 | Pan-Nav1 Na+ channel | IgG1 | IgG2A | AB_2491081 | AB_2750796 |
